# Understanding Populism

**DOI:** 10.1177/09646639231156144

**Published:** 2023-02-16

**Authors:** Jeremy Webber

**Affiliations:** Faculty of Law, University of Victoria, Victoria, BC, Canada

**Keywords:** Populism, democracy, constitutionalism, legal theory, political theory

## Abstract

The diversity of features attributed to populism - and, as a result, the variety of critiques leveled at it - are remarkable. It sometimes seems as though people are using the same terms to address very different phenomena. Is there any distinctive meaning to populism? Is populism inherently anti-democratic or, on the contrary, is it the epitome of democratic practice? What should an engagement with populist movements mean for the theory and practice of democracy? This paper seeks to map the discursive ecosystem that populism determines. It canvasses the phenomena often associated with populism, proposes an interrelated set of concerns that is distinctive to populism, suggests how populism intersects with propensities and affinities with which it is often associated, emphasises the role of growing economic inequality, and suggests responses to populist movements that are grounded in a truly democratic constitutionalism.

## I. Introduction

The meaning of populism is notoriously open, with different commentators fastening upon different features as the essence of the concept. Indeed, populism is sometimes little more than an epithet that critics hurl at political actors who, in the name of the people, pursue policies that the critic thinks unwise. Even in the specialist literature, the range of phenomena attributed to populism is astounding. Populism is indeed a quintessentially contested concept (Mudde and Kaltwasser, 2017: 2-5). In this paper, we unpack that concept, identify its utility in political analysis, consider how it is deployed and how it ought to be deployed, and explore its relationship to democratic theory more generally.

Populism is currently almost always invoked with a negative valence to single out political conduct for criticism. The critics generally argue that this conduct deviates from good democratic practice, although one can be forgiven for thinking that the criticisms are sometimes driven by discomfort with democracy itself, the term then being deployed, as the language of “mob rule” or “demagoguery” was in the past, to attack decisions that have been arrived at by straightforward democratic means (cf Venizelos, 2019). Moreover, one might well argue that, if democracy is fundamentally about collective self-rule, democracy has to be, to some extent, warts and all. Otherwise, how are flesh-and-blood citizens, with their concrete commitments and aspirations, being permitted to rule themselves? Are they only allowed to do so when we, the assessors, think they’ve made an acceptable decision? If that stance is what criticisms of populism come down to, aren’t the people right to rebel?

Indeed, the debate over populism forces us to think harder about the meaning of self-rule, democratic practice, and the constitutional structures that organize and constrain that practice. The debate brings us back to constitutionalism, with that term considered in its broadest sense: not just the legal rules that limit government but also the institutional arrangements and ethical practices that establish government, give government its form, subject it to popular decision-making, and define the people (or peoples) themselves. The debate over populism is therefore intrinsically connected to debates over other essentially contested concepts, notably democracy, citizenship, what it means to be a people, and the rule of law.

The Victoria conference of 6-8 March 2020, out of which this special issue emerged, was designed to explore these issues and, if possible, to clarify them. This essay began its life as a discussion paper for that conference. It canvasses ways in which populism is used, identifying elements that are common; identifying others that are often invoked but that appear to be less universal (elements where the relationship to populism is more affinity or propensity than centrality); and attempting to show how those elements are interrelated. This paper is not intended to define populism. That is (after all) not what one does with an essentially contested concept. But it does seek to sketch the contours that emerge from populism's various elements—or, to mix metaphors, to explore the discursive ecosystem that populism helps to determine. This paper was itself developed discursively, first by a call for input to a group of faculty and graduate students engaged in the Cedar Trees Institute,^1^ then workshopped among a group of some 25 faculty and graduate students on 2 March 2020, and then revisited by the same group on 9 March following the conference. The first-person plural pronouns used in this paper—we, us—generally refer to that group. From time to time, this paper will attribute notable contributions to the participants.

The purpose of this paper is not primarily descriptive. It is to clarify what is at stake in populism for normative political and legal theory—or, better, not for theory but for practice: How should we conduct ourselves as democratic citizens? How should we conceive of the people in whose name democracies govern? What are the appropriate institutions and practices for democratic self-rule?

## II. Populism as an Essentially Contested Concept

If there ever were an “essentially contested concept,” it is populism. The phrase was coined 68 years ago by WB Gallie to understand the role of concepts that are central to particular achievements but where those achievements are internally complex, where the elements that go into the achievement are open to perennial debate, and where the intensity of the debate, sustained in time, is fruitful for the better realization of the achievement (Gallie, 1955). Gallie's four principal examples were art, democracy, social justice, and what it means to adhere to a religion. Such concepts are, he argues, incapable of stable and agreed definition. They epitomize Wittgenstein's insight (1958: para. 67) that definitions are analogous to family resemblances (as Jim Tully emphasized in his input on this paper), in which uses of a word draw upon a range of features that intersect in practice but where no single set of features is shared by all uses. For Wittgenstein, family resemblances are characteristic of all definitions, with speakers drawing upon the overlapping features in their use of terms, generally without controversy. Gallie's essentially contested concepts form a subset of those terms. In essentially contested concepts, controversy is central. The meaning of the terms is vigorously argued over even in the terms’ ordinary use. The contestation accounts in substantial measure for their value. Those employing the concepts express aspirations that they take to be immensely important but that are complex and elusive in their conception, structure, requirements, and realization. The worrying over the terms—the continual arguments over them—drives one's understanding of the complex phenomena they strive to affirm. The terms’ vitality, their fruitfulness, resides in their ability to hold one's attention and spur one's attempts to make sense of them.

“Democracy” is fruitful, for example, because the attempt to give meaning to the ideal of citizens governing themselves forces one to grapple with a set of challenges of foundational importance to political life. The arguments about how to understand and operationalize that ideal illuminate what the aspiration might mean. Those arguments draw participants deeply into explorations of peoplehood, deliberation, consent, solidarity, self-rule, authority, legitimacy. Because of the concept's utility, it is worth probing, identifying its central questions and reflecting upon its entailments and affinities. Doing so helps one to maximize its fruitfulness by sharpening the questions upon which it draws.

In this paper, we consider the debate over populism to be a subset of arguments over democracy. Some commentaries treat populism as though it were essentially authoritarian (Sadurski, 2019: 20). Commentators on “right populisms” (populisms on the right of the political spectrum) often criticize them on grounds that are indistinguishable from criticisms of authoritarian regimes. But, as we will see, the characteristic features of populist movements undeniably engage democratic strategies and concerns (Arditi, 2004: 141-2, 2007: 54-87; Laclau, 2005; Mudde and Kaltwasser, 2012: 17ff; Urbinati, 2014, 2019; Rosanvallon, 2020: 23). Such movements purport to act in the name of “the people”. They appeal to popular sovereignty. They claim that their political programs are anchored within—indeed derive their entire legitimacy from—the will of the people. Populists draw upon an ideal of equal participation in their castigation of elites. Populists may well denounce the existing institutions of democracy but they do so, at least ostensibly, because those institutions do not reflect the people's will. They often argue for mechanisms that, they say, will reflect the people's will more accurately: popular initiatives and referenda; recall of representatives; election of judges; the limitation of judicial review by unelected judges. Such initiatives may be undertaken in bad faith. They may undermine democratic values. They may result in authoritarian government. But it is clear that we are on the terrain of what makes for good democratic government, stable democracies, the advancement of democratic ideals. We are back to arguing over the essentially contested concept of democracy.

The populist challenge forces us to address, yet again, what we mean by democracy and especially how we should do democracy. If authoritarianism were all there were to populism, we might as well drop the label altogether and speak merely of the politics of the right. Indeed, some commentators argue for precisely that outcome, suggesting that the label merely serves to obscure the differences between political movements of the right and left, attaching a pejorative sense to both (eg Mouffe, 2018: 17, although Mouffe seeks to reappropriate the term; Venizelos, 2019; Hopkin, 2020: 6-7; Kraus, 2021; 2-3, 6-7, although Kraus would retain “populism” for illiberal populists). The concept of populism is useful only if it refers to a way of doing politics, one that can be deployed by either the right or the left. For the term to be illuminating, one must acknowledge a distinction between means (political ethics) and ends (political objectives). Populism focuses on the former: on a supposed deformation of democratic practice. Or, to put the point positively, the invocation of populism points toward ways of doing politics that the commentator presumes are much better, much more consonant with democratic ideals, than others (Chatterjee, 2020: 120-21).

This focus on means explains why populist movements are especially relevant to constitutionalism. Constitutions are concerned, above all, with democracy's enabling conditions. They establish the mechanisms, the how, of democratic decision-making. It is no wonder that constitutionalism has become one of the central fields upon which populists and anti-populists contend.

## III. Populism's Elements

Populism is a complex phenomenon. Commentators invoke a number of elements in their descriptions. They often disagree on what elements to include and what weight to attach to them. Indeed, we suggest that populism is best understood in terms of the dynamic interrelationships among elements.

Two approaches shape our account, providing structure to our analysis. First, we distinguish between two categories of elements: 1) core elements—elements that are present in all populist movements and appear to be central to those movements’ populist character; and 2) propensities and affinities—elements that are frequently found in populist movements, indeed may be typical of such movements, but that are not universal and do not appear to be what people mean when they refer to the movements’ populist character. To be clear, we are not suggesting that features in the first category are necessarily more important than those in the second. As will become evident, we consider some items in this paper's category 2 to be very significant, especially important for responses to populism. Indeed, propensities and affinities, even if not universal, may be so prevalent and damaging that they justify concern with elements at the core. That may lead one to guard against them. Propensities matter.

Secondly, our discussion of several of these elements outlines a dynamic spectrum of possibilities. It suggests what drives these possibilities and describes their interrelationships. Commentators generally associate “populism” with the breaching of a particular point along such a spectrum. Different commentators treat different points as the tipping point, which accounts, we believe, for some of the arguments over what constitutes populism. But what is most important is that one considers the full spectrum and reflects upon the dynamics it contains. Remember that arguments over populism are arguments over democracy. Focusing on the spectrum allows us to use populism to illuminate the challenges of democratic constitutionalism, to identify democracy's temptations and pitfalls, to consider responses, and thereby to improve the structures and practices of democracy.

Much of our discussion will seek to identify the point at which we think political conduct becomes corrosive or unhelpful. Our exploration accepts, then, that populism has a negative valence. But note that that negative valence is, in significant measure, stipulative. We are certainly not suggesting that all conduct that others might call populist is negative. We approach this subject with a strong commitment to democratic decision-making as the first principle of contemporary constitutionalism (Webber, 2006). We therefore value, actively, some initiatives that others might term populist. In particular, there are points of interconnection between our orientation and the left populism of Laclau (2005) or Mouffe (2018), though we would argue that our approach has resources for guarding against pathologies to which all populisms, even those of the left, can lead. Our objective is, above all, to explore the preconditions of a healthy, radically democratic order and the ethics appropriate to such an order.

One final preliminary note: The fact that populism's distinctive character lies as much in the interactions among criteria as in a defined set of criteria means that it can appear very differently in different contexts. In the collective that contributed to this paper, we found that we were constantly comparing populisms in different countries, wondering whether we were in fact observing similar phenomena. The principal focus of this conference and special issue were European populisms, especially those of Central and Eastern Europe (CEE), but other regions, especially the Global South, produce populisms that have their own characteristics. Our reflections benefited much from comparisons with those contexts. More comparative work needs to be done (see, eg, Kaltwasser et al., 2017; de la Torre, 2018; Chatterjee, 2020).

## Category 1: Core Elements

We consider there to be four elements the interaction of which characterizes populism.

### 1. Voice of the People

The first and most obvious element is populists’ claim to speak on behalf of the people—not a segment or class but the people in its totality. At first sight, this may seem inclusive, embracing all citizens and even, in some left populisms, humanity at large (eg, Mouffe, 2018: 62ff, although she emphasizes the need for a segment of the people to be treated as the adversary—presumably a constructed adversary as a foil for her constructed people; Kraus, 2021: 5-6, although for Kraus, only some left populisms, which he terms popular republican, are this inclusive). But populism's apparent inclusivity is in tension with the fact that, in any human community, citizens disagree, often profoundly. Populists do not rise above such disagreements. They take sides in them. They then attribute their position to the people as a whole, claiming to be the people's only legitimate representative (Müller 2016: 101) and distinguishing the people's true voice from that of sell-outs and usurpers. In doing so, their claim to speak for the whole ends up advancing a limited and tendentious definition of the people.

This slippage, like many features of populism, has its origin in a conundrum that is foundational to democracy (cf Rosanvallon, 2020: 28-9, 147-51). Any democratic process results in part of the people—typically said to be the majority but for many reasons generally less than an absolute majority—speaking for the whole. Indeed, the very purpose of democratic institutions is to determine what should be society's position against a backdrop of disagreement, ideally through deliberation and persuasion but ultimately through some form of counting heads. The resulting voice is therefore, to a degree, artificial. It is a constructed voice for a public who speak a range of natural voices. Its legitimacy depends upon the extent to which citizens accept the processes by which it has been constructed. And, even with respect to processes, one has to be careful what one means by “acceptance.” Citizens disagree over questions of process as they do over questions of substance (cf. Waldron, 1999). They argue over the distribution of rights of political participation, the institutions, mechanisms and languages by which citizens participate, the level of concurrence required for decisions, the sphere of action that ought to be left to private determination, even the boundaries of the polities themselves. Citizens’ acceptance therefore always involves a measure of acquiescence, of compromise, in processes they consider less than ideal. Indeed, the processes’ legitimacy itself relies upon recursive processes of deliberation and decision. It is through such processes that popular decision-making attains its plausibility. Democratic legitimation therefore shifts attention from the substance of the decisions to the procedures by which the decisions are made (cf Přibáň, 2002).

But note the problem. Citizens are not indifferent to substance. They want their collective decisions to be a projection of their substantive commitments. To the extent that process tends to displace substance, they can be left cold, the result seeming to be thoroughly artificial, the outcome of machinations, not the people's voice (cf Canovan, 2002).

It is this conundrum that populists exploit. They emphasize substantive commitments, not process; organic connection, not mechanism (Bugarič and Kuhelj, 2018: 26-7). They claim to speak for the people directly. Their mandate is derived from their identification with the people's substantive will, not from any artificial construction of that will. Populists claim to possess that will even before they are elected and even when they are voted out of office. They may appeal to standards that derive their meaning from processes—they may claim to speak for “the majority”; they may vaunt their victories in elections or referenda—but those processes ratify substantive commitments whose legitimacy is, in their view, already established, derived directly from the people.^2^ Of course, to some extent, the claim to speak for “the people” is the stock in trade of democrats generally. A common rhetorical strategy is to announce what the people want, in effect exhorting the electorate to agree. Populists tend to step beyond mere exhortation, claiming a more exclusive right to speak for the people. Indeed, when populists are rejected, they may go still farther and challenge the integrity of the processes simply because of that rejection. To head off rejection, they may try to subvert processes, constrain the press, harass NGOs, restrict information, limit debate—all purportedly in service of a higher, substantive, legitimacy.

Their identification with the people consequently projects a bounded definition of the people. This boundedness is true in political terms. The populists claim exclusive possession of the people's political voice; their opponents oppose the people's voice. Populism rejects a pluralist vision of politics (Müller, 2016: 20-1; Mudde and Kaltwasser, 2017: 7-8, 81; Galston, 2018; Bugarič and Kuhelj, 2018: 26-7; Urbinati, 2019). Populists tend to take a winner-take-all approach to political competition (Sadurski, 2019: 3). This also means that they have an inherent tendency to be schismatic and narrowing in the visions of political community they propound. Their emphasis on monopolizing the people's voice has little tolerance for dissent, including dissent within their own party. They continually seek to distinguish the true people from the pretenders, the loyal from the less than loyal, the patriots from the traitors.

It is a mistake to think of this narrowing purely in ideological terms. Many commentators have remarked on populism's ideological thinness or ideological promiscuity (Mudde and Kaltwasser, 2017: 6-7; Mouffe, 2018: 11; Chatterjee, 2020: 93-8). Indeed, ambiguity is necessitated by populism's appeal to an undifferentiated “people”. Too much specificity on questions of policy would draw attention to divisions, among other things endangering the building of a viable political coalition. Populist appeals therefore seek to achieve a difficult balance, emphasizing the unity of the people's voice, distinguishing between the people themselves and those who threaten the people, and yet conveying a conception of the people that is sufficiently ambiguous to constitute a winning coalition yet sufficiently definite to sustain the claim that the populists represent the people themselves (cf Fierman, 2021). As we will see, distinguishing between the true people and “elites” does some of this work, as does, in some movements, a commitment to a charismatic leader. But very often such movements also appeal, sometimes implicitly, sometimes explicitly, to racial, natal, cultural, linguistic, religious, sexual, or other identity grounds.

Doing so allows the movements to harness substantive identification among their followers without foregrounding ideological disagreement. Carl Schmitt (1976: 67), in his argument that distinguishing between friends and enemies constitutes the essence of politics, stipulates that friends and enemies are always “concrete human groupings which fight other concrete human groupings in the name of justice, humanity, order, or peace.” This has a practical logic when politics is conceived in ideologically ambiguous but nevertheless oppositional terms. The commitments upon which populisms draw tend not to be intellectual or, more accurately, are only partially so. They use ascriptive identities as an important ground for their cohesion: Orbán's anti-Semitic dog-whistling; the PiS's nostalgic Catholicism; Modi's Hindu communalism; Trump's softness toward white supremacists; UKIP's exaltation of an idealized and (in selective memory) homogenous Britain during the war. Is the same true of contemporary left populisms? That is less clear—many such populisms certainly don’t want it to be—but their forerunners, the anti-monopolists and populists of the nineteenth century, tended to have narrow conceptions of who counted: they opposed Asian immigrants and, although there were instances of alliances between white populists and African Americans, the former's organizations generally excluded the latter (White, 2011: 298-305, summarized at xxxiv and 513, though White notes exceptions, eg, at 371). Even populists on the left claim to speak for “the people.” To do so successfully, they must, to some extent, take their people as they find them, projecting a collective identity that draws upon the array of identifications held by the people themselves. They may seek to rework and transcend the narrowest of those identities, but any such transcendence will be a work in progress (cf Alinsky, 1946: 99-111, 188-91, 214).

One way in which populism has strong exclusionary tendencies across both left and right is in relation to immigration. The extent to which immigration dominates contemporary populist agendas is remarkable (Betz, 1994: 69-106; Schmidtke, this volume). It is not accidental. Populism trades, above all, on the language of popular sovereignty. That requires at least an implicit conception of the people who are engaging in self-rule. That people gets to determine what its governments do. Governments must act in that people's interests. Such a stance does not preclude generosity toward outsiders but it very often does. It certainly treats them as outsiders. In the case of conflict, the people's interests prevail.

Moreover, this stark division of insiders and outsiders, inherent in the emphasis on self-rule by a specific, physical people, also means that populists tend to be resistant to participation, commentary, or criticism—above all criticism—from those outside the people, including international and supranational organizations. Outsiders have no entitlement to tell the people how to behave.

Now, much of what has been said in this section about populism is characteristic of democracy generally. Any form of democracy places heavy demands on the definition of the people. Democracy needs to have some working conception of who are the bearers of political agency—if nothing else, who gets to vote. Democracy's strong emphasis on equality of participation frequently extends to equality of all citizens, although even there, the simplest and most accessible understanding of equality is sameness of treatment. That commitment is frequently paired with impatience with people who insist on being different, especially if that difference, to persist, needs to be reflected in public policy (such as differences of language, or an Indigenous people's ability to sustain its social, legal, and political order). It is worth remembering that, in settler societies, the colonial governments that tended to be worst in their approach to the Indigenous land question were democratic reformers; the Tories and the imperial authorities were more willing to countenance Indigenous title and institutions (Kirkby, no date). We on the left tend to presume that all progressive causes proceed in lockstep, but there is no doubt that some causes that are deeply cherished are in tension with others, a tension that needs careful resolution. That is true of democracy and differentiation. Nor should one take claims to equality as sameness at face value. An important question is “Sameness to which set of norms, which standard of identity?” That question brings one back to who constitutes the people to whom populists ascribe political agency, a “people” that ends up being a tendentious subset of the democratic citizenry. Think, for example, of the often highly gendered quality of populists’ understanding of the people's voice.

Thus, populism is not simply a departure from democracy. It is a response to—perhaps even an accentuation of—challenges that are baked into democratic self-government. That does not mean that those with an antipathy to populism should cease to be democrats. It does mean that we should think carefully about how we should be democrats.

### 2. Anti-Elitism

Another core quality of populism is its criticism of “elites” (Mudde, 2004: 543; Müller, 2016: 20-1; Mudde and Kaltwasser, 2017: 11-16; Rosanvallon, 2020: 32-5). Populists commonly contrast rule by the people to rule by corrupt elites. This contrast has many facets. It rejects claims, or perceived claims, by individuals or groups to differential political voice. Populists typically are skeptical even of assertions of professional expertise, scientific knowledge, or education generally—think of Trump's championing of the “uneducated”, the Hungarian regime's redirection of education funding to institutions more directly under government control, or the Modi regime's replacement of academic administrators with officers who are tools of the government. This can coincide with a non-rationalist and expressivist conception of truth, in which what counts is the people's holding of a belief rather than that belief's veracity (as János Mécs suggested in our workshops). Moreover, the criticism of elites can have a cultural dimension, expressed in dress (Trump's MAGA caps), in the language of the common people, in religious imagery, in blunt talking, in criticisms of political correctness.

These features are connected to populism's emphasis on democratic egalitarianism.^3^ Whether one likes it or not, each of the features noted above underpins inequality of influence in fact. To someone who lacks the relevant expertise, professional opinions are almost literally unanswerable, at least on their own terms (cf Rosanvallon, 2006: 16). The recipients are, in effect, excluded from the conversation—unless they blow it up. The exclusion is related to education and, in our unequally educated societies, that means generally aligned with social class.^4^ Moreover, the populists’ suspicions are shaped by the realization that expressions of professional opinions, even well-grounded expert knowledge, are not perfectly disconnected from interest. We scholars may struggle for—we should fight for—impartiality and critical discipline in our analyses, but that is a struggle against dispositions of which we may not always be sufficiently aware, as the sociologists of knowledge make clear. Impartiality and self-criticism are virtues that are valuable, they should be affirmed against populist criticism even if their achievement is always imperfect, but that imperfection is clearly one of the mainsprings of populist politics.

The anti-elitism also has a dignitarian dimension to it. Many have commented on the role of dignity in populist appeals (eg Ash, 2018: 24, 2019: 56). There is a strong sense among those susceptible to populist appeals of one's voice not counting, of being disrespected, of historical grievance, of being victimized. That sense is not simply a matter of feelings. It is allied to a perception that government is tone-deaf, that it is not acting upon one's concerns, that it has become too responsive to some people's demands and not at all responsive to others. Again, this attitude crosses left and right populisms.

That said, there is a common irony in populist political movements. They may be anti-elitist in their origins, but, if successful, their leaders become the elite. They frequently end up attracting criticisms not dramatically different from those they leveled at their predecessors. Think of the examples cited above regarding the Hungarian and Indian governments’ intervention in higher education. That is as much the use of governmental power to impose one's personal opinions as the practices they were opposing. Indeed, populists’ manoeuvres are sometimes much more directly so, given their rejection of aspirations to impartiality. Populist governments typically reward their friends, adding more muck to the swamp they once claimed to drain. They rely on processes of decision-making in which they deploy their newfound power. Robert Michels’ iron law of oligarchy (1915) makes its presence felt. It sometimes seems that populist discourse—posing the people against the elites—is self-liquidating.

This irony emphasizes once again that the challenges of populism are the challenges of democracy: how to achieve broad popular participation and translate that participation into decisions that are perceived to be the people's work, even though decisions can never be a matter of unanimous agreement.

### 3. Binary, Moralizing, All-or-Nothing Appeals

Another feature typical of populist movements is the moralizing quality of their discourse (Hawkins, 2010; Mudde and Kaltwasser, 2012: 8-9). Many commentators note that a populist politics tends to be binary, posing the people against the sell-outs. The difference is not a matter of opinion or judgment; it is the difference between rectitude and corruption. Populism eschews consensus and compromise. Why should one compromise with those who are betraying the people? In our conversations, János Mécs suggested that “the moralistic imagination contradicts the very essence of liberal-constitutional representation that treats different political forces neutrally. The inherent inferior-superior quality of the different political forces presupposes some a priori ‘right’ answer that represents anti-pluralism and contradicts the very essence of free political competition.” But note how this comment dramatizes the tension noted above between citizens’ concern with substantive policies and democracy's focus on the institutional determination of a public voice. Citizens themselves are emphatically not neutral. They do believe in right answers. It is just that different citizens believe in different right answers.

The tendency to make moralizing, all-or-nothing appeals follows from populism's claim to possess the substantive voice of the people. It is tough to see how a politics of compromise and consensus-building can survive the assertion of a uniform, unified voice of the people. The Manichean quality of a populist politics can undermine respect for pluralistic mechanisms for government decision-making. If the people's voice is axiomatically single, then institutions premised upon the expression of diverse points of view and their aggregation are misconceived—essentially heretical. Instead of expressing the one true way in simple and direct terms, they blur and complicate. Carl Schmitt expressed this disenchantment with pluralistic democracy well: parliaments replace the true voice of the people with a mechanically derived aggregation which, in the end, represents no-one's true voice (McCormick, 1997: 241). As Ryan Beaton said in our workshops, the antagonistic element of populist politics is therefore intrinsically tied to the next core feature: the distrust of procedures and institutions.

### 4. Distrust of Procedures and Institutions

The fourth of the core elements of populist movements is a marked impatience, an acute distrust, for the institutional mechanisms of government, including parliamentary procedure, hearings, debate, elections that are about deliberation rather than simple affirmation, the rule of law, the separation of powers, the procedures of courts. These are typically seen as little more than obstacles to action. The mechanisms create opportunities—perhaps even design opportunities—through which those who oppose can frustrate the people's will.

Indeed, Wojciech Sadurski (2019: 22-3) considers the dismantling of such institutions to be what is most distinctive about populism. He objects to treating populism as simply a matter of discourse. He notes that many of the purely discursive elements of populism are common in democratic rhetoric generally. For him, populism's distinctive feature is that it actively seeks to eliminate or neutralize the intermediate institutions between the people and the state. Here again we see populists’ impatience with institutional procedures and their claim to cut through those procedures to express the people's will directly. In our conversations, Ryan Beaton noted that populists’ rejection of institutions can occur “relative to particular issues (immigration, economic inequality, climate crises, etc) or relative to a particular message/ideology/value system (nationalism, nativism, ecologism, etc) or relative to the relationship between leader and supporters or the charisma of a leader, or typically some combination of these three. The greater emphasis placed on particular issues, ideology/message, and personal connection, are taken to justify bending or breaking established institutional norms.” Zoltán Pozsár-Szentmiklósy adds: “It is quite controversial that while populism claims the ‘true representation’ of people (and, in that regard, a more perfect realization of people's sovereignty), in reality, populist politics weakens the structures and procedures which support the inclusion of people in politics and public affairs (eg, by weakening the ‘free’ nature of elections as there are no deliberative debates in campaigns).”

There is an important distinction lurking here within populist movements. On one hand, some populists thoroughly reject and seek to deconstruct institutional mechanisms, often because of their constraining effect on executive action: what might be called the populist wrecking-ball. That approach is most often associated with populist leaders—those who wield power. But, on the other hand, much of the popular support for populist movements stems from citizens’ genuine dissatisfaction with the operation of institutions—from motivated criticisms of how those institutions operate and the interests they serve. Citizens’ frustration with institutions can therefore precede and serve as the precondition for the rise of populist movements (Canovan, 1999: 13). One must not confound the two. Criticisms may be well-founded but read as anti-institutional by those who support the status quo. There is no doubt, for example, that contemporary left populism in Europe was, in large measure, a response to decisions of the European Union during the debt crisis (Judis, 2016). Similarly, in nineteenth century United States, the anti-monopoly and populist movements were shaped by the perception—not unreasonable—that governments were acting in collusion with railroads and other industries to siphon public resources into private hands (see eg Goodwyn, 1978; White, 2011).

It would be a mistake, then, to treat the hostility of populists to existing institutions as though populism were simply about the de-institutionalization of government. Populists do chafe at institutional structures, but many engage in credible criticisms that need to be heard, weighed, and their implications considered. Indeed, some populists are genuinely committed to the adoption of what they consider to be better mechanisms for democratic decision-making, referenda being the most obvious example. Even if one does not agree with the detail of populist institutional reforms—even if one opposes the expansion of referenda, for example—the critics may be voicing a dissatisfaction or alienation to which one should attend and respond.

That said, the situation is complicated by the fact that, in populist politics, the bona fide criticisms are sometimes bound tightly together with root-and-branch hostility to institutions. That can be true of supporters of populist political parties. As Ryan Beaton reminded us in our workshops, we should not underestimate the pleasure that people who once were powerless can derive from nihilistic destruction. Populist leaders may invoke bona fide criticisms in service of a broader agenda of institutional subversion. Not only that. Some are manifestly ready to *increase* the dysfunctionality of institutions precisely in order to blow them up. That was a central dimension of the Donald Trump/Steve Bannon strategy. They calculated that any loss of trust in institutions, any acute frustration with their operation, even if induced by their own actions, would redound to their benefit because it would deprive the opposition of these avenues for criticism and reversal. It was a high-risk strategy. It has not yet succeeded. But one can see the logic of the calculation. Even when there is no such conscious strategy, disaffection from established institutions can abet a populist political project. Sadurski (2019: 9) notes that the current Polish government benefits from the public's distrust and disengagement from *all* political parties. If that is true, it is an ironic achievement for a movement that claims to act in the name of the people.

There is a further irony that parallels the one noted under “Anti-Elitism” above. Although populist leaders often express frustration with or seek to subvert institutions, once in power they turn back toward them, frequently remaking those institutions to serve their own ends. In an important sense this is, of course, entirely fair game in a democracy; incoming governments need to be able to pursue the policies that they were elected to enact and this must include adapting institutions to serve those ends. But remarkably often, populist governments go further, seeking to entrench the positions they have now established, creating impediments to the repeal of their policies by a future majority. They assert control over key appointments and regulatory bodies, including those that regulate elections, in order to prevent or delay change (eg, Pap, 2018: 21-22; Sadurski, 2019: 136-49). They politicize the membership of courts or seek to limit the courts’ remit (eg, Pap, 2018: 19-21; Bugarič, 2019: 605-06; Sadurski, 2019: 58-131; Bodnár, 2022: 260-79). They use constitutional amendments and cardinal laws (which require special majorities to change) to insulate key policies against repeal (eg, Venice Commission, 2011: 6-7; Sonnevend et al., 2014; Pap, 2018: 15-17, 23-26; Gárdos-Orosz, 2020: 41). The effect of such actions on popular sovereignty is perverse. In effect, these populists are championing the people's voice only to put themselves in power. The people are not treated as continuously sovereign, able to determine their own governance into the future. This disempowering is cynical: governments that adopt these tactics are in favour of democratic accountability only as long as it serves their interests. But note that it accords closely with the populist claim to embody the people's voice directly and their rejection of the use of procedures and institutions to determine that voice. Indeed, it dramatizes the arrogance of that claim. Populists who take this step know better than the people themselves what the people want. They are willing to protect their rule against actual participation by the people. In so doing, they step outside of any true popular government.

Such a subordination of institutional means to populists’ political ends is related to the stark us-and-them character of populist politics. Means possess an independent value—democratic institutions possess an independent value—if there is a sense of community that transcends the particular controversy. Then it matters that all parties have an opportunity to be heard. A valuing of means also concedes that today's minority might be tomorrow's majority. But if all you care about is the outcome and if you seek to monopolize the definition of that outcome, then process values no longer matter. You subvert institutions when they work against you, and you encase them in concrete when they protect your position.

One might question the long-term durability of this approach. How can populists expect the citizenry to respect the new institutions, those that have been redesigned to frustrate democratic agency into the future? How can they demand respect for process and law when they themselves have subverted them? But, as Keynes said, in the long run we are all dead. More importantly, the populists know that their opponents value process, law, and democracy. Their opponents are unlikely, for good if bittersweet reason, to take up the anti-institutionalist torch. Populists are classic affirmers and disaffirmers.

### 5. Populism as Manipulative, Duplicitous, and Corrupt

Wait! There were only to be four core elements. What is this fifth?

This feature is often advanced in criticisms of populist movements—so often that it might be thought to be essential to our understanding of the phenomenon. Moreover, it is depressingly characteristic of many contemporary populist movements. Should it be considered one of populism's core elements?

No. Certainly, on the right of the populist spectrum, saying one thing and doing another is very common indeed. Moreover, it is clear that a principal objective of many of these leaders—such as Trump in the United States or Orbán in Hungary—has been to use public resources to build up private wealth, often of their friends and cronies. We suspect that many of their most ardent supporters understand that they are doing so, but the supporters’ distrust of institutions (or, for the leading supporters, their cynicism) is sufficiently entrenched that they are content simply that their guy now has the upper hand. It may even be that populist movements are particularly prone to this form of self-dealing. Populist leaders trade, above all, on disenchantment. Especially on the right of the spectrum, they refrain from proposing comprehensive *projets de société.* The very thinness of their ideology and their dismantling of institutional practices open considerable space for self-enrichment.

But we question the utility of such an element for analysis. First, it does not appear to be inherent in populism. The distinctive rhetorical and political strategies of populism—the elements that make the movements recognizably populist—can be pursued in the service of particular ideologies even without dissembling. And of course, a politics of duplicity is hardly unique to populism. Most importantly, such a criticism runs an acute risk of being condescending toward the audience for populist appeals—condescending in a way that involves a further denial of voice to those who may feel, justifiably, that they are excluded from politics. It may treat them as … well, “deplorables”.^5^ Kwame Anthony Appiah criticizes meritocracy for its tendency to smugness, and notes how it can call forth “resentment toward a class defined by its education and its values: the cosmopolitan, degree-laden people who dominate the media, the public culture, and the professions in the US” (Appiah, 2018: 23). Because of this condescension, one can fail to listen to what one's compatriots are saying. One can fail to attend to what underpins their dissatisfaction. The response of anti-populists can be, ironically, a mirror of the exclusionary politics of populists. Indeed, at the limit, it can fall prey to a supercilious anti-democracy.

Indeed, for much the same reason, we reject the tendency of some analysts to treat populist policies as though they were distinctively irrational, merely expedient, and lacking in coherent principle. Arguably, inconsistency and expediency are inherent in any truly democratic politics, for it is the people who call the shots and their opinions are diverse and changeable. Moreover, irrationality and expediency lie substantially in the eye of the beholder. The argument over a policy's merits must lie within the scope of a well-functioning political order; it cannot serve as the standard for legitimate and illegitimate democratic decision-making. Otherwise, one gives substance to populists’ allegation that the technocrats are displacing the people.

Thus, this paper will not focus on a propensity of populist politicians to engage in duplicitous, unprincipled, or incoherent conduct. The one element that, for our purposes, is worth retaining from this critique is the realization that what gives impetus to populist politics may be different for populist leaders and for their supporters. Populism may, in other words, be a different phenomenon when viewed from the perspective of its leaders and that of its followers.

## Category 2: Propensities and Affinities

Already, several propensities and affinities have been noted in the discussion of the core elements. It is necessary now only to list those features—features that are not universally present in populisms but that are very commonly so:

1. A Tendency toward a Narrow and Exclusive Definition of the People
2. A Tendency to Build the Definition of the People around Ascriptive Characteristics, including Racism, Religious Intolerance, the Rejection of Cultural Diversity, Opposition to Sexual Minorities, and a Highly-Gendered Approach to Social Interaction
3. Opposition to Immigration
4. Skepticism of Professional Expertise, Scientific Knowledge, and Education Generally
5. A Sense of Wounded Dignity, Victimhood, Disrespect, or Vulnerability
6. The Fact that Populist Appeals are sometimes Manipulative and Duplicitous

Other propensities have been foreshadowed by the previous discussion:

A Close Connection to Nationalist Politics

The affinity to nationalism follows from populism's emphasis upon expressing the will of the people—almost by definition, then, a specific people, bearers of the right of self-government and to whom the government is accountable. One might imagine that the people might be conceived in plural or compound form, so that populism might invoke multiple levels of “the people” who together form a multinational union or supranational federation. Cantonal populist movements within the Swiss federation might be the best example. Even in the absence of compound membership, the members of one people might respect the attachment of other peoples to their nationality and thereby, in some measure, find common cause with them. But such an open and inclusive conception of political engagement is rare among populisms. Instead, they have a tendency to narrowness precisely because a single people's voice is so integral to their appeal. “Make America Great Again” is the rule. Even the Americans who count are defined in limited terms.

Illiberality

Some commentators have also emphasized that populism is illiberal. This is true of Sadurski (2019: 20), who says, when insisting on the value of the term populism: “We need a language to distinguish between, on the one hand, authoritarianisms that rule by resorting to bare force … and, on the other hand, illiberal regimes that want to be liked or even loved, at least by a significant segment of the electorate.”

We have certainly seen elements that tend toward the illiberal: the narrow definition of the people, especially when that definition relies upon racialized, religious, cultural, or gendered exclusions; the presumption that the people speak with one voice; the subjection of institutions to a purely instrumental logic (the form of illiberality that Sadurski emphasizes). The illiberality is often tempered or targeted, however, especially in left populisms. Sadurski himself notes right populism's attempt to cultivate popular support rather than rely simply on coercion. Moreover, it is quite common for right populism to be strongly committed to one form of “liberalism”: the accumulation of wealth in private hands, although this accumulation is not necessarily entrusted to market mechanisms. For its part, left populism can be (but isn’t always) radically democratic. It is true that populisms of both right and left are almost always resistant to the adjudication of constitutional rights, treating adjudication as a constraint on popular decision-making (Mudde and Kaltwasser, 2017: 81-2, 116-7).

Executive-Dominated Government

Populism's tendency to consider the people's voice to be singular can also coincide with a marked preference for the executive as the dominant branch of government. In republican orders, the fact that the presidency is the only office for which all people vote, the singularity of the president's voice in comparison to the multiplicity and division among members of the legislature, and the capacity of a president to take immediate and hard-edged decisions without a complicated process of debate and approval all combine to make it, in most populisms, the privileged bearer of the people's voice (Rosanvallon, 2020: 47-53; cf Dyzenhaus, 1997: 77; Wilentz, 2005: 399). Democracy is often conceived in plebiscitary form, with the ideal being the whole people coming together to choose their leader. It is no accident that Carl Schmitt, the constitutional champion of the executive, has appeared repeatedly in this account (see also Schmidtke, this volume).

There are, finally, certain propensities that have not been foreshadowed above:

Economic Grievance

There would appear to be no necessary reason why economic grievances should bulk so large in populist politics but they clearly do. The rise of populist parties of both right and left is frequently attributed to economic insecurity, but that explanation is too simple, belied by the rise of populism even in places where incomes have risen well above historical levels (Ash, 2018: 23-4, 2019; Sadurski, 2019: 2, 21). It is not the absolute level of economic welfare that is most significant but the perception that government policy with respect to the economy is unjust—that the economy is being administered in the interest of a privileged few, that burdens and benefits are being distributed unfairly, that a few are being enriched at the expense of the many*.* This can be the result of economic dislocation, but the causes are more varied than that explanation alone suggests. They include the growth of inequality as a result of the adoption of neo-liberal policies; the loss of decently-paying jobs; the inequitable distribution of winners and losers in the CEE's post-Communist transition; the erosion of rural economies; the fact that, in the US government's response to the 2008 financial crisis, large financial institutions were protected while homeowners and small businesses were not; and the effect on southern Europe of the EU's response to the banking crisis.^6^ Economic grievances were equally prominent in historical populist movements, such as those in the United States and Canada which pursued agrarian and working-class interests against the self-dealing of banks, railways, and the latter's allies in government (Laycock, 1990: throughout, esp 12-14; White, 2011: throughout, esp xxxi, 513). Note, in all these examples, the determining role of arguments of justice, of desert. The arguments are grounded in the moral evaluation of economies. They are not simply arguments against globalization, the erosion of national sovereignty, or in favour of national protection (contra Rosanvallon, 2020: 55-62), though each of these things can figure in populist arguments and resonate well with populists’ focus on the national community. Their distinguishing feature is that they seek to subject economic policy to considerations of justice.

Of course, there can be fierce arguments over the justification of such claims. The grievances can, for example, be driven by a group's perception that it is losing its privileged position as a result of the rise of previously subordinated groups. Witness the prominence of race (and its surrogates) in right populist discourse in the United States. Those particular grounds should be rejected (indeed combatted), but one should not dismiss the group's underlying perception of economic injustice too hastily. People can sense that governments are no longer attending to their welfare and yet direct their anger against the wrong targets. They can do so because they have imbued racialized stereotypes, come to rely on racialized comparisons for their own sense of self-worth, live within a culture in which racialized comparisons have most currency, or simply measure their welfare through comparison to others who are proximate rather than to people who are more distant (cf Condon and Wichowsky, 2020: 8-9 and chapters 8 and 9). There may be much better grounds for their sense of economic injustice: the elimination of good jobs, the weakening of unions, the favorable treatment of finance capital, and generally the administration of tax policy so that the wealthy no longer bear their share of social provision (eg, Piketty 2014: 493-514). These developments can drive the growth of inequality and the erosion of self-sufficiency for members of all groups. Indeed, politicians, especially populist politicians, can focus attention on racialized or cultural scapegoats precisely in order to divert their supporters from their inegalitarian policies and from their self-enrichment, as the populist mainstream of the US Republican party, the Orbán government in Hungary, and other populist regimes currently do. For those seeking to counter such policies, it can be a mistake to focus solely on those parties’ racialized or cultural appeals. Doing so can reinforce the view, in one's interlocutors, that racialized conflict is the only concern. It can leave the broader economic inequality unaddressed (López, 2019).

Note that the arguments of populists of both right and left tend to concede, at least tacitly, that the economy ought to be evaluated on the basis of considerations of justice, not the unfettered operation of liberalized markets. Populist governments on the right tend not to redress those injustices in fact, being themselves invested in economic inequality. What is more, they frequently combine their rhetorical embrace of economic grievances with attacks on governments’ capacity to respond, seeking to limit, for example, governments’ ability to raise revenue through taxation. Economic grievances are nevertheless much more prominent in populist political appeals than populism's renowned ideological thinness would suggest.

Rural Bedrock

Finally, populist movements often self-consciously identify themselves with rural areas. This is certainly true on the right but has also been true historically on the left. This identification can be seen as a special case of populist movements’ harnessing of economic grievances, structures of representation that favour rural constituencies, and, in some countries (including Hungary and Poland) the sheer size of the rural population, but, in addition, common aspects of populist appeals are especially well adapted to rural contexts.

Chief among these is populism's ideological ambiguity. The skeptical approach to property rights typical of the socialist left often leaves rural householders, businesses, and artisans cold. At the same time, these groups often believe that they have inferior bargaining power in comparison to urban businesses and would be out-competed in unregulated and international markets. They therefore combine a strong commitment to private property with a desire to constrain what they see as excessive or abusive market power.^7^ The tendency of populist politicians on the right to voice such grievances forcefully, but without seeking fundamental economic transformation, often appeals strongly.

Many residents of rural regions also share a cultural conservativism wedded to an equally conservative nationalism. They consider themselves to be deeply connected to the land and its traditions, stewards of the homeland, less seduced by the cosmopolitanism, agnosticism, laxity, and cultural diversity of the cities. This provides the foundation for a claim to dignity that can be mounted against urban condescension. Right populists typically stand as champions of these positions, mobilizing both the sting of the condescension and the claim to embody a purer vision of the people.

## IV. Ways Forward

That concludes, then, our canvass of the domain of populist discourse. What are the implications for our practice of democratic constitutionalism? Let's begin by returning to the ways in which the challenges of populism are challenges of democracy.

Democracy is about the government that we establish for ourselves. That fact presupposes an answer, at least implicit, to who is that “we.” The most inclusive answers extend membership to all residents of a territory, or at least to all who have a measure of permanent attachment to the territory. But even if such a definition is adopted, in any particular decision some will prevail and some will not. What is the status of those who don’t? Is their voice still the voice of the people or are they now the people's opponents? Populist movements tend toward the latter view.

Moreover, in democracy, the very determination of the people's voice is the product of a complex mechanism, one in which, at each point, choices are made as to who participates, through what avenues, with what scope for deliberation and what standards of assent. Those processes can seem artificial. They *are* artificial, for they always seek to secure a conclusion in the face of what is continued disagreement. Populism seizes upon that artificiality. It claims to be the people's voice unmediated, unconstructed. Of course, any collective position, even the populist position, is constructed. Trump was elected with less than a plurality, let alone a majority, of votes. Even when a government has majority support, that support is itself the product of electoral roles, constituencies, electoral funding, access to media, thresholds of victory, and so on. Even a populist party's own position is the result of an internal process of policy formation. Populism makes hard claims to authenticity of voice, but it does so in the face of the real-world complexities of collective decision-making.

Nevertheless, populists seek to capitalize on the citizenry's frustration with such mechanisms. They commonly do so in four ways. First, they purport to appeal directly to the people, by-passing mechanisms and institutional structures. Second, they sometimes seek to blow up or subvert those structures. It is much easier to claim the people's voice if rival claimants have been silenced. Third, they seek to establish their credentials on substantive rather than procedural grounds. They claim to be the true representatives of the people because they are of the right ethnicity, of the right religion, they are home-grown, they hold the right values, they defend the true people from intrusion and outside criticism. Fourth, any sophisticated populist realizes that mechanisms are indispensable and indeed useful. They therefore use them, cynically, to prevent democratic change once their leadership has been secured.

Populists also seek to capitalize on another conundrum of democratic politics: democracy is based on a principle of equality and yet political participation can never be entirely equal. It can be equal in the formal terms of the right to vote—and, for reasons we’ll review in a moment, the maintenance of that formal equality matters greatly—but in practice the impact of citizens’ voices will be shaped by citizens’ articulateness and cogency (therefore often by their education), by their access to resources to project their voice, by their status in society, by their facility in the national language, by their gender, their religion, the color of their skin, or simply by whether sufficient other citizens agree with them. Populists harness citizens’ perceptions of inequality of influence—or, frequently, their anxiety that they may be losing influence because of the rise of people who are not like them—and mobilize those perceptions in the service of their political project.

How then should one respond? The discussion in this paper points in the following four directions.

First, it should drive us to think more carefully about what kind of “people,” what kind of “nation,” we wish to have.

A centrally defining feature of populist movements is that they claim to act in the name of a people. But peoples are never just given. They too are always, to a degree, made. We need to ask what kind of people our political appeals are creating. As we have seen, populisms often seek to erect sharp divides between the true people and their ostensible opponents on the basis of support for the populist party. The people are those who are faithful. Dissenters are the people's enemies. But what a one-dimensional caricature of a people! It is narrowed and intellectually impoverished, renouncing the very imagination, creation, and debate that must drive a nation's vibrancy. Moreover, it is prone to schism and further narrowing as disagreements emerge internally and the newly disloyal are discarded. The people to which one should aspire ought to treasure, confidently, the vibrancy that comes from diversity of thought and expression.

To be clear, this doesn’t mean that a people should be cherished for a set of abstract principles alone—for elements of their constitutional order that are indistinguishable from any other legally-defined people. Peoples have good reason to cherish the languages in which they have come to express their public and private aspirations, their distinctive traditions of normative debate and discussion, their history of institutional development, their religiously-grounded reflections, their literatures, their musical traditions, their arts. These need not be backward-looking; if the cultures are vibrant, the resources are brought to bear on understanding and navigating the society's current challenges. They need not be pursued in a manner that constrains thought and expression. On the contrary, languages are the vehicles of our thought and expression, and the continuation of the lines of inquiry that are carried by one's own particular language can preserve insights that might otherwise be lost, to the impoverishment of all.

Those who believe in an inclusive sense of community have sometimes been so worried by constraining and aggressive forms of nationalism that they have ceded all valuing of culture, all appreciation of national traditions, to those on the right of the political spectrum, taking refuge in a purely constitutional patriotism. That is not the argument made here. In discussion, the collective that contributed to this paper noted that such an abandonment of the field leaves great scope for those who have a much narrower vision of their societies, among them populists, because it fails to provide any public valuing, expression, or debate in relation to such commitments. The vision of the people in this argument, in contrast, recognizes the value of those commitments, in all their diversity and plurality, but it aspires to engage with them confidently, recognizing the value of putting them to work, testing them, debating them, bringing them into conversation with contrasting traditions. It is a dynamic and outward-looking conception of those elements that make up a people (Webber, 2022).

Second, we need to revitalize and defend the institutions of democracy so that the people themselves—the actual flesh-and-blood citizenry—have means by which they can shape the laws. Moreover, we should ensure that this capacity continues into the future, that it is not eroded, so that a democratic people retains the ability to fashion and refashion its destiny.

This requires the defense of democratic institutions against erosion. The defense must include the protection of the basic right of all citizens to vote, together with measures designed to preserve and vindicate that right: the elimination of exclusions from the franchise; the achievement and maintenance of a definition of citizenship that includes all long-term residents; effective voter registration; vigorous resistance to strategies of voting suppression. It is instructive that each of these elements is under assault by right populists in the world today. Those assaults are another manifestation of populists’ strategy of undermining the institutional structures by which people participate in politics in order to further their claim that they alone speak for the people. Few would suggest that the right to vote, all on its own, is sufficient for a vibrant democratic society. Sometimes those who advocate deliberative democracy or direct action have suggested that the right to vote is not worth much. But that is wrongheaded. If one of the factors that drives populism is the perception of unequal voice, the structural baseline of the equal right to vote, combined with its symbolic prominence, remains crucial. Preserving its integrity sustains the one obvious litmus test, grounded in actual participation, of democratic legitimacy.

But a purely reactive policy of defense is insufficient. The problem is not populist movements’ criticisms of institutions. The problem arises when those movements embrace anti-institutionalism in principle—or to be more accurate, when they pursue the instrumentalization of institutions, or when they squarely commit themselves to the wrecking ball. But often even those populist movements are drawing much of their force from citizens’ disaffection from what the citizens’ consider to be unresponsive, ineffective, or corrupt institutions. The movements channel profoundly democratic dissatisfaction into forms of protest designed to secure a hearing. It would be a great mistake to dismiss those grievances out of hand. On the contrary, we need to cultivate a spirit of scrutiny, experimentation, assessment, responsiveness, and reform in our institutional arrangements. Indeed, on occasion populist movements—especially movements of the left—are experimenting with their own novel institutional forms (see Tully et al., 2022). Those experiments might, at the very least, help us to define more accurately and therefore correct more surely the shortcomings of our existing arrangements. At times they might be worth emulating and developing.

Greater exercise of political choice in decentralized forums—at the municipal level, for example—may be one answer, although it cannot be sufficient. Public concern is most acute with respect to matters that have to be addressed by larger political communities: the achievement of greater economic equality, effective responses to the threat of environmental collapse, the protection of health, Indigenous/non-Indigenous reconciliation, addressing economic dislocation both domestically and internationally, the transformation of energy policy. Moreover, one of the key drivers of populism is the perception of neglect for rural areas, which decentralization can address in only limited fashion. We would love to see governments stimulating debate around methods for addressing these challenges. We admire the policies of public deliberation pursued by the Government of Quebec (in its États généraux) and by Scandinavian countries (La Ligue d’action nationale, 1990; Morin, 1990; Henrikson, Strømsnes and Svedberg, 2019). We are intrigued by the potential of intense policy discussions in microcosm which are then extended to a broader public (such as the deliberative polling of Fishkin, 2018).

Such experimentation creates broader avenues for political participation and reinforces the realization that strong and responsive institutions are necessary to effective self-government. It enables citizens to experience the confrontation of views that occurs in any democratic order and, one hopes, discover ways of making decisions against a backdrop of disagreement. It directly counters the populist anti-institutional mythology. With a little luck, it will build institutions that can retain citizens’ trust.

Note that these measures lie largely within the capacity of democratic governments and a democratic citizenry themselves to achieve. They do not require constraints on democratic decision-making established by courts. Indeed, it is difficult to see how courts could engage in the kind of institutional experimentation contemplated here. Courts have their responsibilities in maintaining the institutional foundations of democratic governance. They can preserve the integrity of the right to vote. They certainly can refrain from decisions that directly undermine that integrity, such as, in the United States, *Citizens United v Federal Election Commission*, 558 US 310 (2010), or *Bush v Gore*, 531 US 98 (2000)*.* They can also protect the structures—of freedom of speech, of association, of the independence of the judiciary, of academic freedom, of parliamentary procedure, of anti-corruption—on which true democratic participation depends. Those bulwarks matter. But it is a mistake to think the courts on their own can protect our societies from an erosion of democratic practices—that they can protect us from our own democratic selves. The challenges of populism are fundamentally the challenges of democracy. They have to met on that ground.

Silvia Suteu has argued that an enabling factor in the turn to populism in the CEE was the fact that the post-Communist constitutions were developed through means that were neither participatory nor deliberative: “They were mostly drafted as elite pacts and have remained in many ways far removed from the societies which they govern.” (Suteu, 2019: 502; cf. Sonnevend et al., 2014). As a result, constitutionalists could not rely upon robust defence from the citizenry, whose knowledge of and attachment to the constitutions were limited. Democratic constitutionalism has to be sustained by democratic means.

Third, the above analysis also suggests the importance of developing an inclusive democratic ethic through democratic practice.

The narrowing of the people typical of populism cannot be countered simply by turning the tables, so that those who are currently disfavoured disregard the others in return. The exclusion of segments of the population from equal citizenship is a driver of populism, not its solution. We suggest, then, that those in government continue to attend carefully to segments of the electorate with whom they will never fully agree, whose votes they will never secure, but to whom they remain accountable and from whom they acquire political insight essential to governing the country as a whole. They should exemplify, in other words, an ethic of inclusive citizenship. They, and we as citizens generally, should model by their practice what it means to be a democratic people, forming part of a political community even when we disagree (cf. Allen, 2004: 149-53).

To be clear, this is not a simplistic reliance on what passes for “bipartisanship” in the US political system, which too often connotes an avoidance of hard-edged decisions and the pursuit of a logrolling politics, where benefits for one are traded off against benefits for another. As will become clear immediately below, decisions that are highly contested are essential. The objective, then, is not to secure unanimous agreement; it is to model respect among those who continue to disagree. That respect may not be reciprocated. In fact, as long as we remain in this era of populist politics we can be certain that it will not be. But what is at stake is our conduct, our imagined political community, not merely that of our opponents.

Fourth, we need to do something serious about economic inequality.

Chantal Mouffe has written of the need to reverse the oligarchization of political decision-making (Mouffe, 2018: esp. 16ff). Although we strongly support greater citizen participation, that particular formulation runs three risks. First, it confuses populism's critique with a diagnosis of populism's causes. The populist critique certainly declares that government is in the control of an oligarchy but in large, mass societies, it is difficult to see how most of the citizenry will not, even after reforms, be one step removed from final decision-making. We suspect that most citizens accept that conclusion. Second, Mouffe's formulation may subject reform to an idealized and unattainable standard, that of direct, continual, political participation in the *polis.* The socialist wag, George Bernard Shaw, is reputed to have said: “Socialism takes too many evenings.” While we strongly support the expansion of avenues for popular participation and apprenticeship in practices of self-government—and have gladly contributed many of our own evenings—Shaw's dictum injects an important note of realism. Third, and most importantly, Mouffe's formulation is misleading for it uses language that invokes the extent of participation to capture a distinction which is, even for Mouffe, fundamentally ideological, having to do with the economic structure of society under the hegemony of neo-liberalism.

We political theorists and constitutional lawyers have approached the requirements of democratic legitimacy too narrowly in pared-down proceduralist and institutional terms. As a result, our constitutionalism has been insufficient. We should refocus on a substantive and much-neglected precondition of democratic legitimacy: the maintenance of some form of equality in the distribution of economic benefits and burdens. Healthy, self-governing polities are buttressed by a rough-and-ready social contract: the sense that citizens bear an adequate balance of the benefits and burdens of citizenship. If burdens and benefits are wildly skewed, trust in political institutions is eroded, and the ability of those institutions to claim the allegiance of their citizens undermined. The equality may not be complete equality of outcomes—indeed never is—but nor is it reducible to the formal equality of the right to vote.

Isn’t that erosion of trust precisely what we see in the age of populist politics? It is that erosion that links economic grievances and populism. We have seen, in each of the societies marked by a rise in populism, a perception that the distribution of economic benefits and burdens is dramatically skewed. The level of inequality worldwide, within countries, rivals that of the Gilded Age.^8^ The prosperity gap between cities and rural districts has been an important driver of rural dissatisfaction. And in the CEE, even if virtually all members of the post-Communist societies are economically better off in absolute terms, the sense of unfairness—of some people scamming the system—is pronounced. It is true that there often appears to be a marked divide between the sources for electoral support for populist politicians and those politicians’ conduct. Populist politicians often appear to be doing their best to entrench the causes of economic inequality to their own advantage. One suspects that they are depending on a variant of the vicious circle sketched above, in which right-wing populists suppress support for the state by making the state *not* work for ordinary folks and thereby maximize the chance that the people will not use the state to rein them in. But there is no doubt that an acute sense of economic injustice is an important element of the populist appeal.

What degree of economic justice is necessary to restore the substantive social contract? There is a sliding scale of economic justice claims. One elementary claim affirms the principle that prerogatives of the state should be used for public purposes and not diverted to private ends. This norm drove the historical populist movements of the late nineteenth century. Today it includes the condemnation of outright corruption, underpins longstanding criticisms of the manner in which assets were privatized in the post-communist transition in the CEE, and accounts for widespread disgust at the sell-off of shares by political leaders in anticipation of the drop in markets caused by COVID-19. A second, only slightly more demanding form is the fair sharing of the burdens of taxation. This is offended by governments’ toleration of income-sheltering in offshore trusts; by international companies’ evasion of taxation by the attribution of income to low-tax jurisdictions; and by the avid participation of some political leaders in the income-hiding exposed in the Panama, Paradise, and Pandora Papers and the continued foot-dragging of virtually all governments in putting a stop to it (no doubt influenced by the interests of their principal donors).^9^ A third, more demanding conception seeks to dampen economic inequality generally through redistributive taxation, so that differences of wealth are kept within tolerable bounds. This third, quintessentially social democratic position preserves the acquisition of property as a spur to economic initiative, together with the freedom of action that individuals and groups obtain from private property, but then taxes and redistributes many of the gains to limit economic inequality. And of course, there remains a fourth category of assertions that aim for a more complete equality of outcomes.

For the purposes of this paper, we remain agnostic as to which of these constitute the ideal form of economic equality. If one's concern is motivated by the rise of populism—or, less narrowly, by the desire that citizens perceive that their political orders are working in their interests, are responsive to their needs, are tolerably fair in their distribution of burdens and benefits, and, in that substantive sense, broadly “democratic”—then what matters is the citizenry's empirical attribution of legitimacy to their governments. The grounds for that attribution are going to be diverse and the necessary equality therefore some aggregation of these grounds (including at the least the first and second and probably the third as well), not some pure ideal.

But that empirical truth does not mean that normative arguments over economic inequality are irrelevant. Citizens’ perceptions are themselves compounds of experience and normative claims. Moreover, sustainable elements of democratic legitimacy may only come into view once their conditions of possibility have been established. It may be, for example, that one has to rebuild confidence in the capacities of governments before governments will be fully trusted to engage in certain legitimacy-producing initiatives. Political leaders may need, then, to aim for objectives that lie beyond those currently supported by their publics, seeking to establish their possibility by progressively rebuilding faith in democratic governance. Fundamentally, it is a mistake to treat normative arguments as existing in isolation from citizens’ attitudes. If one's normative arguments are democratic—if, in other words, they form part of an ensemble that treats collective self-determination as a cardinal virtue in itself—then they must appeal in some way to the wills and aspirations, actual or potential, of the citizenry. Normative arguments, in other words, have the status of well-considered and reflective contributions to democratic decision-making. They do not stand outside democracy.

It is time that we returned to those normative arguments with some seriousness. Many arguments against populism leave the question of economic justice unaddressed. Indeed, they often uncritically champion economic liberalization, affirming political freedom without attending to the conditions that can sustain it. They appeal uncritically to European institutions at a time when those institutions are under fire for the distributional consequences of austerity in southern Europe. They emphasize, above all, the importance of legality and constitutional form. They offer no effective answer to citizens who see growing inequality and the stripping of social protections and rebel. No wonder those citizens turn to politicians who at least pretend to care.

Economic equality is not something that constitutional adjudication can achieve. The best adjudication can do is correct discrete anomalies and nullify exceptional impositions upon the poor and the underprivileged. But who said that constitutionalism is limited to what courts can achieve? So many of us, legal scholars and political scientists alike, now think of constitutions in quintessentially legal terms. We have all become constitutional lawyers. If we believe in democratic constitutionalism, we need to return to a more comprehensive vision of the conditions of democracy.

## Notes

1. Participants included Ryan Beaton, Renáta Bedő, Eszter Bodnár, Akshaya Chandani, Keith Cherry, David Gill, Arvind Kumar, Eva Linde, Ratana Ly, Rebeca Macias Gimenez, Fazila Mat, János Mécs, Zehra Munshi, Rebeccah Nelems, Pablo Ouziel, Benjamín Perrier, Songkrant Pongboonjun, Zoltán Pozsár-Szentmiklósy, Himaloya Saha, Beate Schmidtke, Oliver Schmidtke, Adrianne Stacey, James Tully, Esteban Vallejo Toledo, Mary Anne Vallianatos, Jeremy Webber, and Claire Wood. The Cedar Trees Institute was created by a number of scholars (both faculty and students), centred at the University of Victoria but extending internationally, to stimulate study and reflection upon the futures of democracy, drawing upon the plurality of democratic action and imagination that exists inside and outside states. For another product of that group, see Tully et al. (2022).

2. Rosanvallon (2020: 39-41) therefore takes populists’ supposed commitment to referenda too seriously. As he himself notes in his historical review (102-3), referenda are invoked by many populists as a mode of symbolic affirmation – “un rituel de l’unanimité” – rather than the determination of the people’s will. He is right, however, to note populists’ emphasis on the substantive unity of the people and that unity’s frequent expression in a single charismatic representative (Rosanvallon, 2020: 43-53). Something similar might be said of the differences (limited though they are) between this paper’s argument and that of Urbinati (2019). Treating populism as “a new form of representative government” can lead Urbinati to give too much credit to populist claims to seek to and actually represent a majority of citizens, so that her critique leans heavily on populists’ failure to accept the *alternation* of majorities. Populists do want to win, but they are often content to do so by undermining the very structures that underpin majority representation in the first place (electoral machinery; free speech; parliamentary debate; accountability). That said, Urbinati certainly does consider populists’ democratic claims to be “disfigured.”

3. Condon and Wichowsky (2020: 187-89) rightly emphasize the currency, on all sides of US politics, of arguments “for the little guy” against elite privilege. Müller (2016: 1-7) makes a similar point regarding democratic political rhetoric generally.

4. For a thought-provoking empirical exploration of the complex interrelations of class, education, and political participation in Britain, see Ansell and Gingrich (2022).

5. This, of course, was the term then-candidate Hillary Clinton used, during the 2016 presidential campaign, to refer to half of Donald Trump’s supporters: “Read Hillary Clinton’s ‘Basket of Deplorables” Remarks about Donald Trump’s Supporters,” *Time* (10 September 2016): https://time.com/4486502/hillary-clinton-basket-of-deplorables-transcript/.

6. This list is too comprehensive to allow for full citations. For accounts that focus on neo-liberalism, see Judis (2016), Mouffe (2018), Hopkin (2020).

7. This too has historical antecedents. It was a prominent characteristic of the North American populist movements of the late 19^th^ century (Goodwyn, 1978: XI-XVI).

8. See Chancel and Piketty (2021) and the databases and working papers available on the World Inequality Database: https://wid.world/.

9. The focus on fairness in taxation had proto-populist antecedents (eg Tomlins, 1993: 3, quoting William Manning’s emphasis on proportionality in the burden of taxation).
